# Metabolomic profiles of hepatocellular carcinoma in a European prospective cohort

**DOI:** 10.1186/s12916-015-0462-9

**Published:** 2015-09-23

**Authors:** Anne Fages, Talita Duarte-Salles, Magdalena Stepien, Pietro Ferrari, Veronika Fedirko, Clément Pontoizeau, Antonia Trichopoulou, Krasimira Aleksandrova, Anne Tjønneland, Anja Olsen, Françoise Clavel-Chapelon, Marie-Christine Boutron-Ruault, Gianluca Severi, Rudolf Kaaks, Tilman Kuhn, Anna Floegel, Heiner Boeing, Pagona Lagiou, Christina Bamia, Dimitrios Trichopoulos, Domenico Palli, Valeria Pala, Salvatore Panico, Rosario Tumino, Paolo Vineis, H. Bas Bueno-de-Mesquita, Petra H. Peeters, Elisabete Weiderpass, Antonio Agudo, Esther Molina-Montes, José María Huerta, Eva Ardanaz, Miren Dorronsoro, Klas Sjöberg, Bodil Ohlsson, Kay-Tee Khaw, Nick Wareham, Ruth C. Travis, Julie A. Schmidt, Amanda Cross, Marc Gunter, Elio Riboli, Augustin Scalbert, Isabelle Romieu, Benedicte Elena-Herrmann, Mazda Jenab

**Affiliations:** Institut des Sciences Analytiques, Centre de RMN à très hauts champs, CNRS/ENS Lyon/UCB Lyon-1, Université de Lyon, 5 rue de la Doua, 69100 Villeurbanne, France; International Agency for Research on Cancer (IARC-WHO), Lyon, France; Department of Epidemiology, Rollins School of Public Health, Winship Cancer Institute, Emory University, Atlanta, GA USA; Hellenic Health Foundation, Alexandroupoleos 23, GR-115 27 Athens, Greece; Bureau of Epidemiologic Research, Academy of Athens, Kaisareias 13, GR-115 27 Athens, Greece; Department of Epidemiology, German Institute of Human Nutrition (DIfE), Potsdam-Rehbrücke, Germany; Diet, Genes and Environment, Danish Cancer Society Research Center, Strandboulevarden 49, DK 2100 Copenhagen, Denmark; INSERM, Centre for Research in Epidemiology and Population Health (CESP), U1018, Nutrition, Hormones and Women’s Health Team, F-94805 Villejuif, France; Université Paris Sud, UMRS 1018, F-94805 Villejuif, France; Institut Gustave Roussy, F-94805 Villejuif, France; Human Genetics Foundation (HuGeF), Torino, Italy; Department of Cancer Epidemiology, German Cancer Research Centre, Heidelberg, Germany; Department of Hygiene, Epidemiology, and Medical Statistics, University of Athens Medical School, 75 M. Asias, Goudi, GR-115 27 Athens, Greece; Department of Epidemiology, Harvard School of Public Health, 677 Huntington Avenue, Boston, MA 02115 USA; Molecular and Nutritional Epidemiology Unit, Cancer Research and Prevention Institute – ISPO, Florence, Italy; Epidemiology and Prevention Unit, Fondazione IRCCS Istituto Nazionale dei Tumori, Via Venezian 1, 20133 Milano, Italy; Dipartimento di Medicina Clinica e Chirurgia, Federico II University, Naples, Italy; Cancer Registry and Histopathology Unit, “Civic - M.P. Arezzo” Hospital, Ragusa, Italy; MRC-PHE Centre for Environment and Health, School of Public Health, Imperial College London, London, UK; Department for Determinants of Chronic Diseases (DCD), National Institute for Public Health and the Environment (RIVM), Bilthoven, The Netherlands; Department of Gastroenterology and Hepatology, University Medical Centre, Utrecht, The Netherlands; Department of Epidemiology and Biostatistics, School of Public Health, Imperial College London, London, UK; Department of Social & Preventive Medicine, Faculty of Medicine, University of Malaya, Kuala Lumpur, Malaysia; Department of Epidemiology, Julius Center for Health Sciences and Primary Care, University Medical Center Utrecht, Utrecht, The Netherlands; Department of Community Medicine, Faculty of Health Sciences, University of Tromsø, The Arctic University of Norway, Tromsø, Norway; Department of Research, Cancer Registry of Norway, Oslo, Norway; Department of Medical Epidemiology and Biostatistics, Karolinska Institutet, Stockholm, Sweden; Samfundet Folkhälsan, Helsinki, Finland; Unit of Nutrition and Cancer, IDIBELL, Catalan Institute of Oncology-ICO, L’Hospitalet de Llobregat, Barcelona, 08908 Spain; Escuela Andaluza de Salud Pública, Instituto de Investigación Biosanitaria ibs.GRANADA, Hospitales Universitarios de Granada/Universidad de Granada, Granada, Spain; CIBER Epidemiología y Salud Pública (CIBERESP), Madrid, Spain; Department of Epidemiology, Murcia Regional Health Council, IMIB-Arrixaca, Murcia, Spain; Navarre Public Health Institute, Pamplona, Spain; Public Health Direction and Biodonostia CIBERESP, Basque Regional Health Department, San Sebastian, Spain; Department of Clinical Sciences, Lund University, Malmö, Sweden; Department of Gastroenterology and Nutrition, Skåne University Hospital, Malmö, Sweden; Department of Clinical Sciences, Division of Internal Medicine, Skåne University Hospital, Lund University, Malmö, Sweden; University of Cambridge School of Clinical Medicine, Clinical Gerontology Unit, Addenbrooke’s Hospital, Cambridge, UK; MRC Epidemiology Unit, University of Cambridge, Cambridge, UK; Cancer Epidemiology Unit, Nuffield Department of Population Health, University of Oxford, Oxford, UK

**Keywords:** Epidemiology, European Prospective Investigation into Cancer and Nutrition, Hepatocellular carcinoma, Liver cancer, Metabolomics, Nuclear magnetic resonance

## Abstract

**Background:**

Hepatocellular carcinoma (HCC), the most prevalent form of liver cancer, is difficult to diagnose and has limited treatment options with a low survival rate. Aside from a few key risk factors, such as hepatitis, high alcohol consumption, smoking, obesity, and diabetes, there is incomplete etiologic understanding of the disease and little progress in identification of early risk biomarkers.

**Methods:**

To address these aspects, an untargeted nuclear magnetic resonance metabolomic approach was applied to pre-diagnostic serum samples obtained from first incident, primary HCC cases (n = 114) and matched controls (n = 222) identified from amongst the participants of a large European prospective cohort.

**Results:**

A metabolic pattern associated with HCC risk comprised of perturbations in fatty acid oxidation and amino acid, lipid, and carbohydrate metabolism was observed. Sixteen metabolites of either endogenous or exogenous origin were found to be significantly associated with HCC risk. The influence of hepatitis infection and potential liver damage was assessed, and further analyses were made to distinguish patterns of early or later diagnosis.

**Conclusion:**

Our results show clear metabolic alterations from early stages of HCC development with application for better etiologic understanding, prevention, and early detection of this increasingly common cancer.

**Electronic supplementary material:**

The online version of this article (doi:10.1186/s12916-015-0462-9) contains supplementary material, which is available to authorized users.

## Background

Liver cancer is the sixth most commonly diagnosed cancer and the second leading cause of cancer death worldwide [1]. Hepatocellular carcinoma (HCC), the most frequent type of liver cancer, is primarily associated with chronic hepatitis B (HBV) and C (HCV) infections and aflatoxin exposure [2], while other major risk factors include obesity, type 2 diabetes, tobacco smoking, and heavy alcohol drinking [3–5]. HCC is highly malignant, usually diagnosed at late stages, and often has a poor prognosis with limited treatment options [6]. The late diagnosis and consequent poor survival associated with the disease are often attributed to its lack of pathognomonic symptoms and limitations of diagnostic modalities. Improving both the understanding of HCC etiology and the early detection of the disease is an important first step towards the design of effective prevention strategies aimed at early diagnosis and reduction of HCC incidence. A valuable tool toward these goals is the analysis of bio-samples from prospective cohort studies, where healthy participants are enrolled and followed over time for the appearance of various diseases. Since HCC development implies alterations in the metabolic functions of the liver and, in a majority of cases, progresses from pre-cancerous lesions through to cirrhosis and cancer, it is conceivable that metabolic changes may be detected from the very early stages of the disease, long prior to clinical diagnosis. Thus, metabolomics may serve as a valuable tool for the identification of biomarkers for early detection of HCC.

Metabolomics is a powerful high-throughput approach that relies on state of the art analytical methods, such as nuclear magnetic resonance (NMR), to identify metabolic signatures or biomarkers associated with homeostasis perturbations [7]. Metabolomic strategies play an increasingly important role in clinical and observational studies, in the hope that they will offer new perspectives not only in understanding the processes of disease development, but also for identification of diagnostic/prognostic markers and targeted healthcare [8]. Indeed, several recent studies have leveraged metabolite profiling to provide new insights into pathological processes pertaining to cancer, heart disease, or diabetes mellitus [9–15]. Although a number of metabolomic-based approaches have been applied to HCC, they have either been largely based on traditional case–control designs, high risk patient groups (e.g. hepatitis infection, cirrhosis, or other chronic liver diseases), non-Western populations where traditional HCC risk factors predominate, or on tumor tissues [16–31]. However, there is currently very little information derived from prospective settings where biological samples have been collected prior to disease diagnosis [32–34].

In this study, we investigated whether metabolic differences could be detected between HCC cases and matched controls derived from a prospective cohort study using serum samples collected prior to diagnosis. A NMR-based metabolomic approach was applied to a case–control study nested within a large, multi-center prospective cohort.

## Methods

### Study design

The present study is based on a case–control study nested within the European Prospective Investigation into Cancer and Nutrition (EPIC) cohort, a multicenter prospective study designed to investigate the association between diet, lifestyle, and environmental factors and the incidence of various types of cancer and other chronic diseases. The rationale, detailed study design, and methods have been previously detailed [35]. Briefly, diet and lifestyle data were collected at recruitment from approximately 520,000 men and women aged 35–85 years enrolled between 1992 and 2000 in 23 centers from 10 Western European countries (Denmark, France, Germany, Greece, Italy, Norway, Spain, Sweden, the Netherlands, and the United Kingdom) [35]. The study subjects were recruited from the general population, except for France (women who were members of a health insurance scheme for state school employees), Naples and Norway (women only), Utrecht and Florence (women attending breast cancer screening), and subsamples of the Oxford “Health Conscious” sub-cohort (vegetarians) and the Italian and Spanish cohorts (mainly members of blood donor associations).

### Ethics

The EPIC cohort in general, and this study in particular, have received approval from the Ethics Committee of the International Agency for Research on Cancer as well as the ethics review boards of individual EPIC centers. EPIC participants provided written consent for the use of their blood samples and all data.

### Blood sample collection

Blood samples were collected using standardized methods at recruitment from most participants and are stored at IARC (Lyon, France) in liquid nitrogen at –196 °C for all countries except Denmark (−150 °C, nitrogen vapor) and Sweden (−80 °C, freezers) where samples are stored locally [35].

### Cancer and vital status assessment

Vital status during follow-up (98.5 % complete) was assessed by record linkage with regional and/or national mortality registries in all countries except Germany and Greece, where follow-up was actively reported by study subjects or their next-of-kin. Cancer incidence was determined through record linkage with population-based regional cancer registries (Denmark, Italy, the Netherlands, Norway, Spain, Sweden, and the United Kingdom) or via a combination of methods, including the use of health insurance records, contacts with cancer and pathology registries, and active follow-up through study subjects and their next-of-kin (France, Germany, Greece). For the present study, the dates of follow-up for cancer incidence and vital status are complete up to end of 2006.

### The HCC nested case–control study

#### Ascertainment of cases

HCC cases were defined as tumor in the liver (C22.0) according to the 10th Revision of the International Statistical Classification of Diseases, Injury and Causes of Death. For each HCC case identified, the histology, methods used to diagnose the cancer, and α-fetoprotein (AFP) levels were reviewed to exclude metastatic cases or other types of primary liver cancers.

#### The nested case–control study

The design of the nested case–control study has been previously described in detail [36]. Briefly, 125 HCC cases with available blood samples at baseline were identified between participants’ recruitment and 2006. For each case, two controls were selected by incidence density sampling from all cohort members alive and free of cancer (except non-melanoma skin cancer), and matched by age at blood collection (±1 year), sex, study center, date (±2 months) and time of the day at blood collection (±3 h), and fasting status at blood collection (<3/3–6/>6 h). Women were additionally matched by menopausal status (pre-/peri-/postmenopausal) and hormone replacement therapy use at time of blood collection (yes/no). Participants with insufficient remaining blood sample for NMR analyses were excluded (N_cases_ = 11). For six cases, only one eligible control was available for each case. Therefore, the final sample size for the present analysis included 114 HCC cases and 222 matched controls.

### Serum sample analysis

#### Laboratory assays: HBV/HCV infection, biomarkers of liver function and AFP

HBV and HCV seropositivity were detected in serum samples using the ARCHITECT HBsAg and anti-HCV chemiluminescent microparticle immunoassays (CMIAs; Abbott Diagnostics, France): HBsAg-positive when ≥0.05 IU/mL and HCV-positive when the ratio of sample relative light units to cutoff relative light units was ≥1 in two measurements [36]. Biochemical markers of hepatic injury, including albumin, total bilirubin, alanine aminotransferase (ALT), aspartate aminotransferase (AST), gamma-glutamyltransferase (GGT), and liver-specific alkaline phosphatase (AP) were measured on the ARCHITECT c Systems™ (Abbott Diagnostics) using standard protocols. The normal ranges were: albumin, 35–50 g/L; total bilirubin, 3.4–20.5 mmol/L; ALT, <55 U/L; AST, 5–34 U/L; GGT, 12–64 U/L (men) and 9–36 U/L (women); and AP, 40–150 U/L. A liver function score was calculated from concentrations of albumin, total bilirubin, ALT, AST, GGT, and AP, each contributing 1 point when outside of the normal range [37]. The liver score was categorized as no liver damage (liver score 0), probable liver damage (liver score 1–2), and likely liver damage (liver score ≥3). Additionally, the concentration of serum AFP, which is currently a pre-diagnostic biomarker for HCC, was measured in blood using the ARCHITECT AFP kit. The laboratory analyses were performed at the Centre de Biologie République, Lyon, France [36].

#### NMR metabolomic data acquisition

**Fig. 1:**
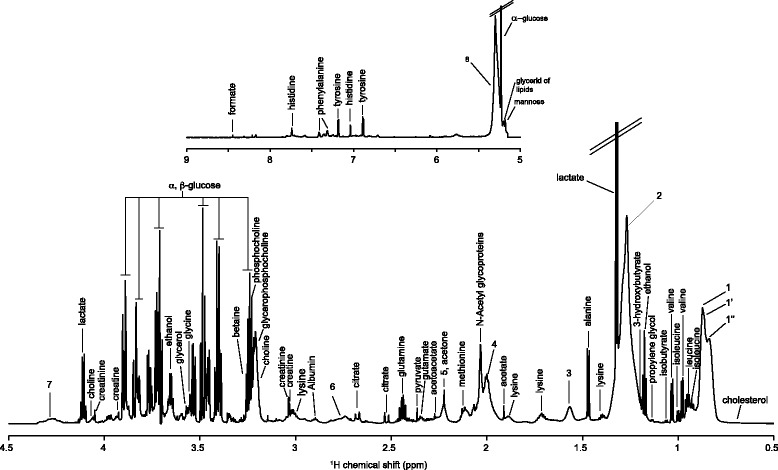
Mean ^1^H Carr-Purcell-Meiboom-Gill NMR spectrum of serum samples with metabolite assignment. 1, C**H**_3_ bond of lipids, mainly VLDL; 1’, C**H**_3_ bond of lipids, mainly LDL; 1”, C**H**_3_ bond of lipids, mainly HDL; 2, C**H**_2_ bond of lipids; 3, C**H**_2_-CH_2_-COOC bond of lipids; 4, C**H**_2_-CH = bond of lipids; 5, CH_2_-C**H**_2_-COOC bond of lipids; 6, =CH-C**H**_2_-CH = bond of lipids; 7, Lipid O-C**H**_2_; 8, C**H** = C**H** bond of lipids

Serum samples (200 μL) were processed according to standard procedures for NMR metabolomic measurement [38]. One-dimensional ^1^H Carr-Purcell-Meiboom-Gill (CPMG) and Nuclear Overhauser effect spectroscopy (NOESY) NMR spectra were recorded for each serum sample on a Bruker Avance III spectrometer operating at 800.15 MHz ^1^H NMR frequency. Additional two-dimensional NMR spectra were recorded on a set of representative samples (one control and one case) to achieve assignment of the NMR signals observed in the ^1^H one-dimensional fingerprints to metabolites. The measured chemical shifts were compared to reference shifts of pure compounds using the HMDB [39], MMCB [40], and ChenomX NMR Suite (Chenomx Inc., Edmonton, Canada) databases. Figure 1 shows the mean CPMG spectrum with metabolite assignments. The detailed list of the 44 annotated metabolites is provided in Additional file 1: Table S1. NMR signals arising from lipids enabled the quantification of unsaturated lipids in the serum (signal at 5.28 ppm, resonance of -C**H** = C**H**- from unsaturated lipids) as well as terminal lipids methyls corresponding to several classes of lipoproteins: very-low-density lipoproteins (VLDL; δ 0.86 ppm), low-density lipoproteins (LDL; δ 0.84 ppm), and high-density lipoproteins (HDL; δ 0.82 ppm). After processing and calibration, each 1D NMR spectrum was reduced into bins of 0.001 ppm width over a chemical shift range of 0.5–9 ppm using the AMIX software (Bruker GmbH, Rheinstetten, Germany), giving a total number of 8,500 NMR variables.

All NMR analyses were performed blindly with respect to case/control status. Further details on sample preparation, NMR data acquisition, and spectra processing are available in Additional file 1.

### Statistical analysis

#### Orthogonal partial least-square (O-PLS)

O-PLS [41] analyses were conducted in order to build predictive sample classification models based on whole CPMG or NOESY NMR spectra to discriminate between HCC cases and controls, by relating the 8,500 NMR variables to case/control status. Results were visualized on score plots corresponding to sample projection onto the predictive axis and the first orthogonal component of the model. The metabolic signature discriminating HCC cases from controls was visualized by the corresponding loading plot. The optimal number of orthogonal components for building O-PLS models was selected using a 7-fold cross validation procedure. The associated R^2^ and Q^2^ parameters were calculated as a measure of the “goodness of fit and prediction”, i.e. the explained and predicted variances, respectively. The robustness of O-PLS models was further validated using permutations (1000 times) under the null hypothesis; for each permutated case/control labels, R^2^ and Q^2^ values were obtained and compared to the original ones, their decrease indicating the good quality of the model [42].

#### Metabolite paired difference analysis

The statistical recoupling of variables [43] procedure was first applied to reduce the 8,500 NMR variables into 285 intelligent buckets, or clusters of NMR variables, that correspond to reconstructions of peak entities. ANOVA models were then carried out on each of the 285 clusters of variables by modelling the case–control set by means of a random effect variable to account for the matching design of the study in ANOVA mixed-effect models. To correct for multiple testing, *q* values were determined using the Benjamini-Hochberg procedure [44] to control the false discovery rate with a threshold of 0.05. In this way, 96 clusters of NMR variables were found to be significantly associated with HCC outcome. Significant clusters of variables corresponding to different peaks of the same metabolite (based on the metabolite identification reported above) were combined into a single variable by summing up the bins intensities taking into consideration the number of homolog protons in the signal resonance. This procedure resulted in a list of 23 combined clusters of variables, 16 of which corresponded to distinct metabolite or lipid classes and were retained for further analyses, while five corresponded to other signals from mixed classes of lipids and two corresponded to the superimposition of signals from different metabolites.

#### Conditional logistic regression (CLR)

CLR models were used to quantify the associations between the 16 metabolites selected as described above and HCC risk by computing odds ratios (OR) and 95 % confidence intervals (95 % CIs). The metabolites were modeled as continuous variables with the OR corresponding to one standard deviation increase in metabolic intensity. CLR models were run conditioned on the matching factors (referred to as crude), and after adjustment for potential confounding variables (referred to as multivariable), i.e. body mass index (continuous), smoking status (current smokers, non-smokers, former smokers, unknown), lifetime alcohol drinking pattern (never drinkers, former drinkers, drinkers only at recruitment, lifetime drinkers), level of alcohol consumption at recruitment (g/d; continuous), serum-clot contact time (≤1 d or >1 d; a value that corresponds to the time between blood collection and blood centrifugation [45]), physical activity (inactive, moderately inactive, moderately active, active, missing), educational status (primary school, secondary school, professional school, longer education, unknown; as a proxy variable for socioeconomic status), and waist circumference (cm). The multivariable models for serum ethanol concentration were not adjusted for level of alcohol consumption at recruitment. For all metabolites, an additional CLR model with further adjustment for liver function score was also run.

#### Receiver operating characteristics (ROC)

ROC curves and corresponding area under the curve (AUC) were generated for several models including the AFP concentration, the liver function score, the multivariate metabolic profile using both the score values from the O-PLS classification model (referred as O-PLS score), and the cross-validated predicted-Y values (referred as O-PLS CV status) as well as a combination between the O-PLS CV status and AFP or the liver score. Combinations of the variables were obtained by summing up the O-PLS CV status with either AFP or the liver score after normalization of each variable to one unit variance. The specificity, sensitivity, and accuracy were obtained from the optimal cut-off point that corresponded to the minimal distance to the ideal point.

#### Subgroup analyses

Analyses stratified by hepatitis infection status (37 HCC cases Hep^+^, 77 HCC cases Hep^–^), by liver function score (34 HCC cases with no liver damage, 80 HCC cases with probable to certain liver damage), by years between blood collection and cancer diagnosis with a cut-off at 2 years (22 HCC cases diagnosed <2 years, 92 HCC cases diagnosed ≥2 years from blood collection) were also conducted. In the grouping of cases diagnosed <2 years, the small sample size prevented model stability upon multivariable adjustment. Thus, only crude CLR models were run for this subgroup.

The analyses were performed using SIMCA-P 12 (Umetrics, Umeå, Sweden), MATLAB (The MathWorks Inc., Natick, MA) routines developed in-house, and R software [46] using the packages ‘splines’ and ‘survival’.

## Results

Baseline characteristics of the study participants are summarized in Table 1. The median follow-up time between blood collection and HCC diagnosis (lag time) was 4.8 years. Serum blood samples of HCC cases were more likely to test positive for HBV or HCV infections (32.5 % vs. 3.2 % in the controls), and to have altered liver function as indicated by high liver function score (36.8 % vs. 14.4 % for probable liver damage and 33.3 % vs. 0.9 % for likely liver damage for cases vs. controls, respectively).

**Table 1:** Characteristics of study participants in the EPIC study. Values refer to either median (minimum – maximum range) or number (percentage)

	HCC cases	Matched controls
Characteristics	(n = 114)^a^	(n = 222)
Gender		
Men	79 (69.3 %)	153 (68.9 %)
Women	35 (30.7 %)	69 (31.1 %)
Age at blood collection (years)	60.6 (46.1–77)	60.2 (45.7–77)
Age at diagnosis (years)	65.3 (47.6–86.4)	
Lag time (years)^b^	4.8 (0.01–13.3)	
Body mass index (kg/m^2^)	27.7 (19.5–43.4)	26.5 (17.2–40.2)
Waist circumference (cm)	95 (68.5–140)	92.2 (64–130)
Smoking status		
Never	30 (26.3 %)	94 (42.3 %)
Former	39 (34.2 %)	86 (38.7 %)
Current smoker	44 (38.6 %)	41 (18.5 %)
Unknown	1 (0.9 %)	1 (0.5 %)
Serum-clot contact time^c^		
≤1 day	84 (73.7 %)	169 (76.1 %)
>1 day	30 (26.3 %)	53 (23.9 %)
Educational status		
Primary school/None	61 (53.5 %)	106 (47.7 %)
Secondary school	4 (3.5 %)	23 (10.4 %)
Technical/professional school	30 (26.3 %)	45 (20.3 %)
Longer education	18 (15.8 %)	44 (19.8 %)
Unknown	1 (0.9 %)	4 (1.8 %)
Physical activity		
Inactive	10 (8.8 %)	29 (13.1 %)
Moderately inactive	36 (31.6 %)	71 (32 %)
Moderately active	57 (50 %)	100 (45 %)
Active	11 (9.6 %)	22 (9.9 %)
Lifetime alcohol drinking pattern		
Never drinkers	10 (8.8 %)	17 (7.7 %)
Former drinkers	17 (14.9 %)	3 (1.4 %)
Drinkers only at recruitment	17 (14.9 %)	43 (19.4 %)
Lifetime drinkers	70 (61.4 %)	159 (71.6 %)
Alcohol intake at recruitment (g/day)	7.5 (0–141.3)	8.8 (0–108.6)
HBV/HCV status		
No	77 (67.5 %)	215 (96.8 %)
Yes	37 (32.5 %)	7 (3.2 %)
Liver function score^d^		
No	34 (29.8 %)	188 (84.7 %)
Probable	42 (36.8 %)	32 (14.4 %)
Likely	38 (33.3 %)	2 (0.9 %)
AFP (ng/mL)	5.3 (0–18780)	3.25 (0–20.8)

^a^Distribution of HCC cancer cases across EPIC countries was 29 for Denmark, 20 for Germany, 11 for Greece, 19 for Italy, 4 for the Netherlands, 7 for Spain, 16 for Sweden, and 8 for the United Kingdom

^b^Follow-up time between blood collection and HCC diagnosis

^c^Serum-clot contact time refers to the time between blood collection and blood centrifugation for serum isolation

^d^A categorical liver function score describing liver damage was constructed based on values of albumin (<35 g/L), total bilirubin (>20.5 μmol/L), AST (>34 U/L), ALT (>55 U/L), GGT (men >64 U/L, women >36 U/L), and AP (>150 U/L), each contributing 1 point when outside of the normal range

**Fig. 2:**
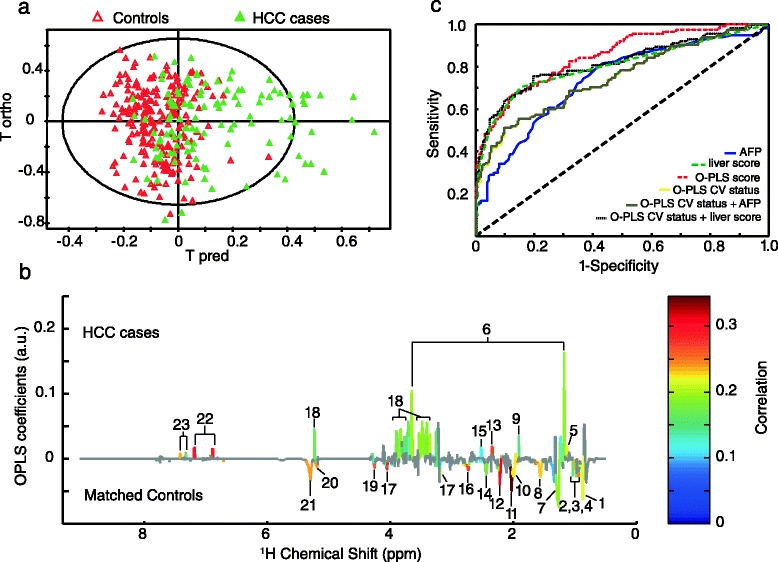
NMR Metabolomic discrimination between HCC cases (n = 114) and matched controls (n = 222) based on ^1^H Carr-Purcell-Meiboom-Gill NMR data. (**a**) Orthogonal partial least-square (O-PLS) score plot of NMR spectra, R^2^ = 35 %, Q^2^ = 21 %. (**b**) O-PLS metabolic signature colored according to the correlation between NMR variables and case–control status after significance to ANOVA tests followed by Benjamini-Hochberg multiple correction (non-significant NMR variables are colored in grey). The validation of the model is presented in Additional file 1: Figure S1a. 1, C**H**_3_ bond of lipids mainly very-low-density lipoproteins; 2, Leucine; 3, Isoleucine; 4, Valine; 5, Propylene glycol; 6, Ethanol; 7, C**H**_2_ bond of lipids; 8, C**H**_2_-CH_2_-COOC bond of lipids; 9, Acetate; 10, C**H**_2_-CH = bond of lipids; 11, N-acetyl glycoproteins; 12, Acetone and CH_2_-C**H**_2_-COOC bond of lipids; 13, Glutamate; 14, Glutamine; 15, Citrate; 16, =CH-C**H**_2_-CH = bond of lipids; 17, Choline; 18, Glucose; 19, Lipid O-C**H**_2_; 20, Mannose and lipids; 21, C**H** = C**H** bond of lipids; 22, Tyrosine; 23 Phenylalanine. An equivalent metabolic signature obtained from ^1^H NOESY NMR data is presented in Additional file 1: Figure S1b. (**c**) ROC analyses including AFP, liver function score, O-PLS score, O-PLS cross-validated (CV) status, and a combination between O-PLS CV status and AFP or liver function score. The ROC of O-PLS CV status and the combination of O-PLS CV status and AFP overlap. The characteristics of each model are presented in Table 3

The O-PLS analysis presented in Fig. 2a shows a metabolic profile discriminating between HCC cases and the matched controls (R^2^ = 35 %, Q^2^ = 21 %). The metabolic signature (Fig. 2b) associated with HCC occurrence presented (1) higher levels in the aromatic amino acids (AAA) tyrosine and phenylalanine, glutamate, acetate, citrate, glucose, propylene glycol, and ethanol; (2) lower levels in unsaturated lipids and VLDL, N-acetyl glycoproteins, choline, glutamine, acetone, mannose and the branched-chain amino-acids (BCAA) valine, leucine, and isoleucine levels, compared to the control group. The corresponding *P* values, *q* values, and fold changes of the metabolites are presented in Table 2. The ROC analyses (Fig. 2c) of the metabolic signature (O-PLS score) and of the cross-validated data (O-PLS CV status) presented an AUC of 85 % and 74 %, respectively (Table 3). The ROC parameters obtained from AFP compared to the combination of O-PLS CV status with AFP were increased after combining the variables (AUC 73 % vs. 75 %, specificity 65.3 % vs. 80.6 %, sensitivity 71.9 % vs. 75.4 %, accuracy 67.5 % vs. 78.9 %). Multivariable adjusted CLR models showed that AAA (per 1-SD), tyrosine (OR = 2.46; 95 % CI, 1.65–3.6), phenylalanine (OR = 2.07; 95 % CI, 1.40–3.06), glutamate (OR = 2.44; 95 % CI, 1.54–3.87), citrate (OR = 1.76; 95 % CI, 1.22–2.54), glucose (OR = 1.67; 95 % CI, 1.19–2.35), and propylene glycol (OR = 2.20; 95 % CI, 1.06–4.60) were associated with a statistically significant higher HCC risk. In contrast, BCAA, leucine (OR = 0.60; 95 % CI, 0.43–0.85), isoleucine (OR = 0.72; 95 % CI, 0.53–0.98), choline (OR = 0.45; 95 % CI, 0.31–0.65), N-acetyl glycoproteins (OR = 0.46; 95 % CI, 0.32–0.67), unsaturated lipids (OR = 0.36; 95 % CI, 0.21–0.63), and VLDL (OR = 0.52; 95 % CI, 0.36–0.74) were inversely associated with HCC risk (Table 4).

**Table 2:** *P* values, *q* values, and fold differences of metabolites observed to be statistically significantly different between HCC cases (n = 114) and matched controls (n = 222)

	HCC cases vs. matched controls		
Metabolites	*P* value^a^	*q* value^b^	Fold difference
Lipids = CH-C**H**_2_-CH=	9 × 10^-11^	3 × 10^-08^	0.84
N-acetyl glycoproteins	3 × 10^-10^	4 × 10^-08^	0.90
Unspecific^c,d^	4 × 10^-10^	4 × 10^-08^	0.82
Glutamate	4 × 10^-09^	3 × 10^-07^	1.22
Tyrosine	1 × 10^-08^	7 × 10^-07^	1.24
Choline	2 × 10^-08^	7 × 10^-07^	0.82
Lipids O-C**H**_2_	1 × 10^-07^	3 × 10^-06^	0.84
Phenylalanine	2 × 10^-07^	5 × 10^-06^	1.23
Unspecific^c,e^	4 × 10^-07^	9 × 10^-06^	0.84
Leucine	2 × 10^-06^	3 × 10^-05^	0.91
Lipids C**H**_2_-CH_2_-COOC	2 × 10^-06^	3 × 10^-05^	0.82
Unsaturated lipids^f^	2 × 10^-06^	3 × 10^-05^	0.86
Isoleucine	2 × 10^-05^	2 × 10^-04^	0.90
Lipids C**H**_2_-CH=	2 × 10^-05^	2 × 10^-04^	0.94
C**H**_3_ bond of lipids (VLDL)^g^	2 × 10^-05^	2 × 10^-04^	0.87
Propylene glycol	7 × 10^-05^	6 × 10^-04^	1.45
Lipids (C**H**_2_)n	1 × 10^-04^	8 × 10^-04^	0.88
Ethanol	3 × 10^-04^	2 × 10^-03^	1.65
Glutamine	7 × 10^-04^	4 × 10^-03^	0.90
Glucose	8 × 10^-04^	4 × 10^-03^	1.10
Valine	3 × 10^-03^	1 × 10^-02^	0.93
Acetate	5 × 10^-03^	2 × 10^-02^	1.24
Citrate	1 × 10^-02^	4 × 10^-02^	1.08

^a^ *P* value was obtained from ANOVA mixed-effects models

^b^ *q* value corresponds to the value obtained after Benjamini-Hochberg correction for multiple testing

^c^Multiple assignments for the same cluster of variables due to spectral overlap

^d^Acetone and CH_2_-C**H**_2_-COOC of bound lipids

^e^Mannose and glyceride of lipids

^f^Unsaturated lipids as estimated from the C**H** = C**H** signal of lipids

^g^Part of terminal lipids methyl signal corresponding to VLDLs

**Table 3:** AUC, specificity, sensitivity, and accuracy of the ROC models (in %)

	All				Cases diagnosed <2 years				Cases diagnosed ≥2 years			
	AUC	Spe	Sen	Acc	AUC	Spe	Sen	Acc	AUC	Spe	Sen	Acc
AFP^a^	73	65.3	71.9	67.5	81	79	77.3	78.5	71	60.9	74	65.3
Liver function score^b^	80	84.7	70.2	79.8	79	88.4	68.2	81.5	80	83.8	70.6	79.3
O-PLS score^c^	85	81.5	71.1	78.1	93	83.7	86.4	84.6	79	74.9	68.5	72.7
O-PLS CV status^d^	74	68	68.4	68.1	82	100	63.6	87.7	71	68.7	67.4	68.3
O-PLS CV status + AFP^e^	75	80.6	75.4	78.9	82	100	63.6	87.7	73	70.9	70.6	70.8
O-PLS CV status + liver score^f^	82	68	68.4	68.1	84	86	77.3	83	80	83.8	70.7	79.3

AUC, Area under the curve; Spe, Specificity; Sen, Sensitivity; Acc, Accuracy of the model

^a^Model based on the serum AFP concentration

^b^Model based on the liver function score

^c^Model based on the score of the O-PLS analysis

^d^Model based on the cross-validated (CV) case/control status of the O-PLS analysis

^e^Combination between the CV status of the O-PLS and the AFP variable

^f^Combination between the CV status of the O-PLS and the liver function score

**Table 4:** Odds ratios [OR (95 % confidence intervals)] of HCC risk by serum metabolites

	Crude^a^	Multivariable^b^	Multivariable + liver function score^c^	Multivariable for non-hepatitis infected cases^d^	Crude <2 years follow-up^e^	Crude ≥2 years follow-up^f^	Multivariable ≥2 years follow-up^g^
	114 cases	114 cases	114 cases	77 cases	22 cases	92 cases	92 cases
N-acetyl glycoproteins	0.47 (0.36–0.62)***	0.46 (0.32–0.67)***	0.76 (0.49–1.17)	0.74 (0.49–1.14)	0.64 (0.39–1.05)	0.43 (0.31–0.60)***	0.35 (0.21–0.59)***
Glutamate	2.37 (1.64–3.41)***	2.44 (1.54–3.87)***	1.46 (0.80–2.63)	2.06 (1.18–3.61)*	4.87 (1.26–18.82)*	2.05 (1.43–2.94)***	2.13 (1.30–3.49)**
Tyrosine	2.00 (1.51–2.65)***	2.46 (1.65–3.66)***	1.58 (0.98–2.56)	1.42 (0.83–2.43)	3.90 (1.50–10.14)**	1.83 (1.36–2.47)***	2.23 (1.41–3.54)***
Choline	0.37 (0.27–0.51)***	0.45 (0.31–0.65)***	0.83 (0.52–1.32)	0.68 (0.42–1.12)	0.30 (0.13–0.66)**	0.39 (0.27–0.56)***	0.45 (0.29–0.71)***
Phenylalanine	1.83 (1.37–2.44)***	2.07 (1.40–3.06)***	1.75 (1.04–2.94)*	1.59 (0.94–2.67)	3.59 (1.29–10)*	1.62 (1.20–2.19)**	1.64 (1.07–2.54)*
Leucine	0.54 (0.41–0.71)***	0.60 (0.43–0.85)**	0.67 (0.43–1.05)	0.68 (0.43–1.09)	0.80 (0.48–1.33)	0.47 (0.34–0.66)***	0.51 (0.33–0.80)**
Unsaturated lipids	0.29 (0.18–0.46)***	0.36 (0.21–0.63)***	0.77 (0.41–1.44)	0.59 (0.29–1.23)	0.16 (0.04–0.60)**	0.33 (0.20–0.53)***	0.46 (0.24–0.84)*
Isoleucine	0.64 (0.49–0.83)***	0.72 (0.53–0.98)*	0.84 (0.56–1.26)	0.76 (0.50–1.15)	0.84 (0.50–1.42)	0.59 (0.44–0.80)***	0.66 (0.45–0.96)*
VLDL	0.58 (0.45–0.76)***	0.52 (0.36–0.74)***	0.77 (0.51–1.15)	0.74 (0.45–1.20)	0.30 (0.12–0.72)**	0.64 (0.49–0.85)**	0.58 (0.39–0.87)**
Propylene glycol	3.07 (1.58–5.97)***	2.20 (1.06–4.60)*	1.00 (0.59–1.69)	1.27 (0.73–2.21)	727.9 (1.56–340468)*	2.11 (1.24–3.58)**	1.46 (0.83–2.55)
Ethanol^h^	1.76 (1.13–2.74)*	1.46 (0.97–2.21)	1.02 (0.69–1.51)	1.10 (0.74–1.65)	126.1 (1.3–12227)*	1.66 (1.09–2.53)*	1.40 (0.95–2.08)
Glutamine	0.66 (0.51–0.84)**	0.75 (0.54–1.03)	0.96 (0.64–1.44)	0.56 (0.34–0.92)*	0.60 (0.30–1.15)	0.67 (0.51–0.88)**	0.74 (0.51–1.07)
Glucose	1.53 (1.18–1.99)**	1.67 (1.19–2.35)**	1.28 (0.86–1.92)	1.24 (0.80–1.92)	1.95 (0.98–3.88)	1.47 (1.10–1.96)**	1.77 (1.16–2.69)**
Valine	0.69 (0.54–0.88)**	0.82 (0.61–1.11)	0.90 (0.61–1.34)	0.78 (0.51–1.18)	0.75 (0.44–1.27)	0.68 (0.52–0.89)**	0.79 (0.55–1.13)
Acetate	1.40 (1.08–1.82)*	1.20 (0.90–1.60)	0.92 (0.58–1.46)	1.01 (0.63–1.61)	3.04 (0.45–20.69)	1.32 (1.01–1.75)*	1.12 (0.76–1.63)
Citrate	1.36 (1.06–1.76)*	1.76 (1.22–2.54)**	1.88 (1.14–3.11)*	1.15 (0.65–2.01)	2.19 (1.10–4.35)*	1.22 (0.93–1.61)	1.37 (0.91–2.06)

*P value <0.05; **P value <0.01; ***P value <0.001

^a^Model 1, Crude OR based on logistic regression conditioned on matching factors (sex, age, date and time of blood collection, fasting status, and menopausal status and use of hormones)

^b^Model 2, OR based on multivariable CLR adjusted for smoking status, ethanol at recruitment, lifetime alcohol, educational status, physical activity, body mass index, serum-clot contact time, and waist circumference

^c^Model 3, as in model 2 and additionally adjusted for liver function score

^d^Model 4, as model 2 but obtained on HCC cases free of HBV/HCV infection and their matched controls (n = 224)

^e^Model 5, Crude OR obtained on HCC cases diagnosed <2 years after blood collection and their matched controls

^f^Model 6, Crude OR obtained on HCC cases diagnosed ≥2 years after blood collection and their matched controls

^g^Model 7, as in model 2 but obtained on HCC cases diagnosed ≥2 years after blood collection and their matched controls

^h^OR of ethanol from multivariable CLR model are obtained after adjustment for variables as mentioned for model 2 expect for lifetime alcohol

**Fig. 3:**
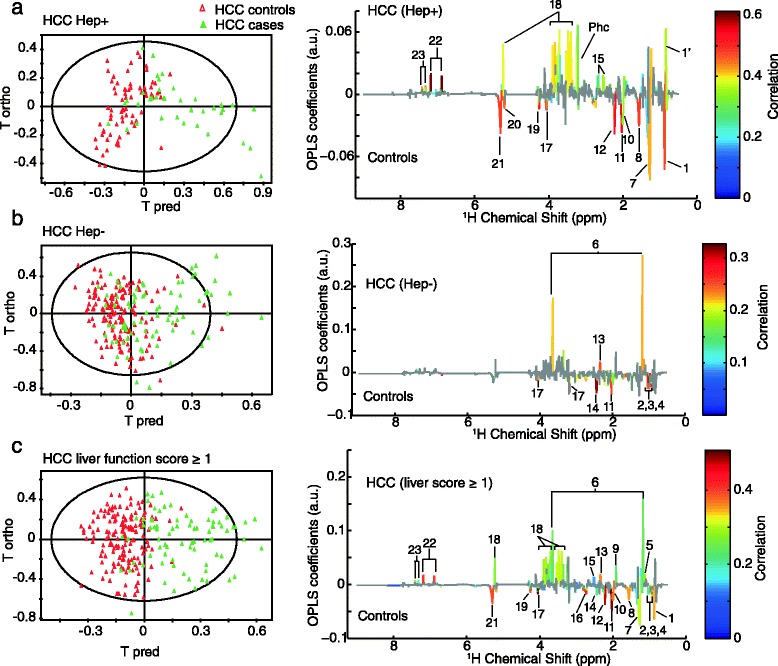
Stratification of the analysis by hepatitis infection status and liver function score. (**a**) O-PLS score plot including HCC cases infected by HBV or HCV (n = 37) and matched controls (n = 72), R^2^ = 45 % and Q^2^ = 34 %, and the metabolic signature. (**b**) O-PLS score plot including HCC cases with HBV/HCV free (n = 77) and matched controls (n = 150), R^2^ = 28 % and Q^2^ = 12 %, and the metabolic signature colored for correlation after significance to ANOVA tests (Benjamini-Hochberg multiple corrected). (**c**) O-PLS score plot including HCC cases with liver function score ≥1 (n = 80) and matched controls (n = 155), R^2^ = 58 % and Q^2^ = 43 %, and the metabolic signature colored for correlation after significance to ANOVA tests (Benjamini-Hochberg multiple corrected). The validations of the O-PLS models are presented in Additional file 1: Figure S2. 1, C**H**_3_ bond of lipids mainly VLDL; 1’, C**H**_3_ bond of lipids, mainly LDL; 2, Leucine; 3, Isoleucine; 4, Valine; 5, Propylene glycol; 6, Ethanol; 7, C**H**_2_ bond of lipids; 8, C**H**_2_-CH_2_-COOC bond of lipids; 9, Acetate; 10, CH_2_-CH = bond of lipids; 11, N-acetyl glycoproteins; 12, Acetone and CH_2_-C**H**_2_-COOC bond of lipids; 13, Glutamate; 14, Glutamine; 15, citrate; 16 = CH-C**H**_2_-CH = bond of lipids; 17, Choline; 18, Glucose; 19, Lipid O-C**H**_2_; 20, mannose and lipids; 21, C**H** = C**H** bond of lipids; 22, Tyrosine; 23 Phenylalanine. Phc, Phosphocholine

The O-PLS analyses stratified by hepatitis infection status of the cases (Fig. 3a,b) presented distinct metabolic signatures from hepatitis-infected HCC cases (R^2^ = 45 %, Q^2^ = 34 %) and hepatitis-free HCC cases (R^2^ = 28 %, Q^2^ = 12 %). Hepatitis-infected HCC cases presented (1) higher levels of AAA, glucose, and citrate and (2) lower VLDL and unsaturated lipids levels, while on the other hand HCC hepatitis-free cases were characterized by (1) higher levels in ethanol and glutamate and (2) lower levels in glutamine, BCAA, and choline. In hepatitis-free HCC cases, the risk associations of glutamine (OR = 0.56; 95 % CI, 0.34–0.92) and glutamate (OR = 2.06; 95 % CI, 1.18–3.61) were significantly different from matched controls (Table 4).

Figure 3c shows O-PLS subgroup analysis of HCC cases with abnormal liver function (score ≥1). A robust model was obtained (R^2^ = 58 %, Q^2^ = 43 %) and the metabolic signature was similar to that including all samples (Fig. 2b). However, no significant model was obtained from HCC cases with a normal liver function (score = 0) only (data not shown). Table 4 shows results of multivariable CLR additionally adjusted for liver function score for which only citrate (OR = 1.88; 95 % CI, 1.14–3.11) and phenylalanine (OR = 1.75; 95 % CI, 1.04–2.94) remained significantly associated with HCC risk.

**Fig. 4:**
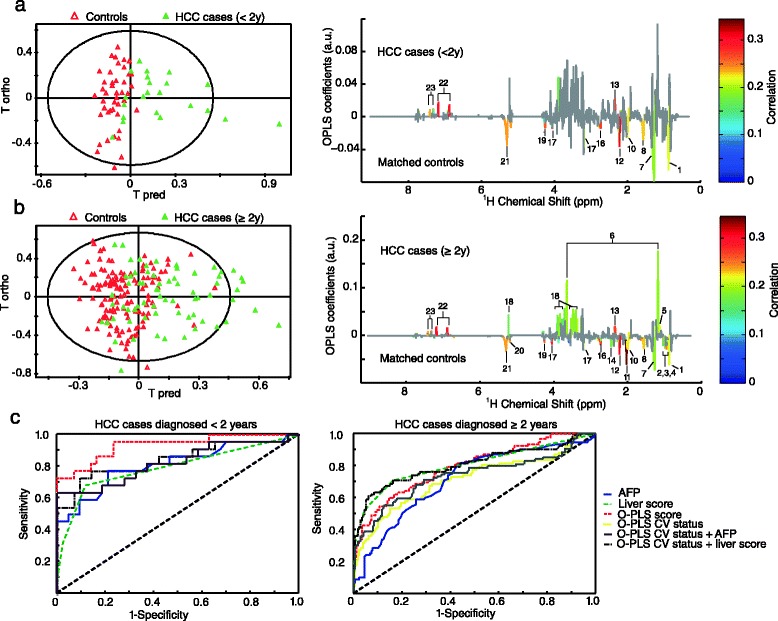
Analyses stratified by the interval between recruitment into the EPIC cohort and clinical diagnosis of HCC (<2 years after recruitment vs. ≥2 years after recruitment). (**a**) O-PLS score plot including HCC cases that were diagnosed <2 years (n = 22) after blood collection and matched controls (n = 43), R^2^ = 45 % and Q^2^ = 33 %, and the metabolic signature colored for correlation after significance to ANOVA tests (Benjamini-Hochberg multiple corrected). (**b**) O-PLS score plot including HCC cases that were diagnosed ≥2 years after blood collection (n = 92) and their matched controls (n = 179), R^2^ = 27 % and Q^2^ = 16 %, and the metabolic signature colored for correlation after significance to ANOVA tests (Benjamini-Hochberg multiple corrected). (**c**) ROC analyses for each stratified group including AFP, liver function score, O-PLS score, O-PLS cross validated (CV) status, and a combination between O-PLS CV status and AFP or liver function score. The ROC curves of the O-PLS CV status and the O-PLS CV status + AFP are almost overlapped for the ROC analysis performed on cases diagnosed <2 years. The characteristics of each model are presented in Table 3. The validations of the O-PLS models are presented in Additional file 1: Figure S3. 1, C**H**_3_ bond of lipids mainly VLDL; 2, Leucine; 3, Isoleucine; 4, Valine; 5, Propylene glycol; 6, Ethanol; 7, C**H**_2_ bond of lipids; 8, C**H**_2_-CH_2_-COOC bond of lipids; 9, Acetate; 10, CH_2_-CH = bond of lipids; 11, N-acetyl glycoproteins; 12, Acetone and CH_2_-C**H**_2_-COOC bond of lipids; 13, Glutamate; 14, Glutamine; 15, Citrate; 16 = CH-C**H**_2_-CH = bond of lipids; 17, Choline; 18, Glucose; 19, Lipid O-C**H**_2_; 20, Mannose and lipids; 21, C**H** = C**H** bond of lipids; 22, Tyrosine; 23, Phenylalanine

Figure 4 presents the O-PLS and ROC analyses stratified by lag time between blood collection and diagnosis. The metabolic signature of HCC cases diagnosed within 2 years after blood collection is characterized by (1) higher levels in AAA and glutamate, and (2) lower levels in unsaturated lipids and choline while in addition, the metabolic signature of HCC diagnosed later (≥2 years) presented (1) higher levels in glucose, ethanol, and propylene glycol and (2) lower levels in BCAA and N-acetyl glycoproteins. Among the cases diagnosed <2 years from recruitment, the AUC of ROC curves from the O-PLS metabolic signature and from O-PLS CV data were 93 % and 82 %, respectively (Fig. 4c).

Higher ROC parameters (Table 3) were found for O-PLS CV status compared to AFP and the liver score (O-PLS CV status vs. AFP, liver score: AUC 82 % vs. 81 %, 79 %; specificity 100 % vs. 79 %, 88.4 %; sensitivity 63.6 % vs. 77.3 %, 68.2 %; accuracy 87.7 % vs. 78.5 %, 81.5 %). However, the parameters did not improve after combining O-PLS CV status with AFP while they were slightly improved after combining O-PLS CV status with the liver score (AUC 84 %; specificity 86 %; sensitivity 77.3 %; accuracy 83 %). ROC analysis of the cases diagnosed ≥2 years from recruitment showed an AUC of 79 % for the O-PLS metabolic signature and 71 % for the O-PLS CV status. Combining the O-PLS CV status with AFP improved the ROC parameters in comparison to AFP alone or O-PLS CV alone (O-PLS CV status + AFP vs. AFP, O-PLS CV: AUC: 73 % vs. 71 %, 71 %; specificity: 70.9 % vs. 60.9 %, 68.7 %; sensitivity: 70.6 % vs. 74 %, 67.4 %; accuracy: 70.8 % vs. 65.3 %, 68.3 %). However, the best model was obtained from the liver score (AUC 80 %, specificity 83.8 %, sensitivity 70.6 %, accuracy 79.3 %).

Findings for subgroup CLR analyses of individual metabolites by lag time of diagnosis from recruitment show a significant HCC risk association for citrate in cases diagnosed <2 years from recruitment (OR = 2.19; 95 % CI, 1.10–4.35), while for cases diagnosed ≥2 years from recruitment significant HCC risk associations were observed for glucose (OR = 1.47; 95 % CI, 1.10–1.96), acetate (OR = 1.32; 95 % CI, 1.01–1.75), N-acetyl glycoproteins (OR = 0.43; 95 % CI, 0.31–0.60), BCAA (valine OR = 0.68; 95 % CI, 0.52–0.89); leucine (OR = 0.47; 95 % CI, 0.34–0.66); isoleucine (OR = 0.59; 95 % CI, 0.44–0.80), and glutamine (OR = 0.67; 95 % CI, 0.51–0.88) (Table 4).

## Discussion

This study is, to the best of our knowledge, the first NMR metabolomic analysis based on subjects from a prospective cohort study on Western European populations for epidemiology of liver cancer. We have identified a number of metabolites that differed between HCC cases and corresponding matched controls. As concerns the specificity of these associations, we note that an analogous study was conducted in parallel on extrahepatic/intrahepatic bile duct carcinomas without providing any significant results (data not shown). We also note that the impact of long-term storage of EPIC samples as well as other potential sources of systematic variations of the metabolic profiles has been thoroughly detailed earlier [45].

O-PLS analysis showed a clear discrimination between cases and controls with somewhat different metabolomic profiles with respect to the length of time from blood collection to diagnosis, hepatitis infection status, and liver function. Importantly, this study showed that consideration of metabolomic profiles can improve HCC diagnosis beyond that provided by AFP and liver enzyme levels, which are currently the most common HCC biomarkers often applied in clinical practice.

The liver is central for the metabolism of carbohydrates, fats and proteins, and also plays key roles in detoxification and hormone production. Thus, a degree of metabolic dysregulation would be expected with liver diseases, particularly HCC. For this reason, the application of metabolomic technologies may be able to provide some insight into the etiology and mechanisms of HCC and, possibly, the identification of early diagnostic biomarkers or biomarker patterns characteristic of cancer at this anatomical site.

To date, three NMR or NMR/mass spectrometry, serum-based metabolomic studies have been conducted looking specifically at HCC [20, 23, 24]. All three case–control studies were based on sera collected from HCC cases post-diagnosis. The comparison group in one of the studies was hepatitis-infected subjects [20], while that of the others were cirrhotic patients [23, 24]. The studies identified potential (1) impairment of the tricarboxylic acid cycle, increased lipid catabolism, and elevation of essential amino acids [20], and (2) defects on ammonium detoxification and increased fatty acid beta-oxidation [24] in HCC. The fundamental design differences with the present study are that the latter is based on prospectively identified HCC cases, such that metabolomic profiles are likely indicative of pre-diagnostic changes, and that the matched control subjects were cancer-free cohort participants. The key metabolic alterations observed are related to changes in amino acid, polyunsaturated lipid, acetate, and citrate metabolism, among the 16 individual metabolites highlighted here. Because our study is nested within the prospective EPIC cohort, which has detailed information on dietary and lifestyle factors and measured anthropometry, we were able to make statistical adjustments for many important confounding variables such as smoking status, alcohol consumption and habits, physical activity, educational attainment (as a proxy marker for socioeconomic status), body mass index, and waist circumference.

Of particular note is our observation of a 0.82-fold reduction in choline in HCC cases (Table 2), meaning a significant inverse HCC risk association for this compound (Table 4; OR = 0.45; 95 % CI, 0.31–0.65). In animal studies, choline deficiency has been shown to cause liver damage, oxidative stress, and spontaneous liver cancer [47–49]. In human studies, HCC has been associated with a down regulation in choline metabolism [25].

Also interesting is our identification of circulating ethanol as a strong HCC risk factor, alcohol being a major lifestyle risk factor for this disease. We also observed a shift, in terms of fold difference between HCC cases and their matched controls, from glutamine to glutamate, indicating a possible defect in ammonium detoxification [50], as also observed in the study by Nahon et al. [24]. It is of interest that Nahon et al. [24] observed this shift comparing HCC cases to cirrhotic controls, while our findings indicate that this important change may actually be present for some time prior to diagnosis. In the study by Gao et al. [20], higher levels of AAA were associated with liver cirrhosis and HCC, together with lower levels of BCAA, choline, and unsaturated lipids. The same changes were observed suggesting an important alteration of amino acid and lipid metabolism in the progression to HCC.

An interesting observation in the present study was a strong, significant positive HCC risk association for the exogenous metabolite propylene glycol. Identification of propylene glycol in human serum is not uncommon [51], and it is thought to derive largely from pharmaceutical use since it is widely used as a solvent in many intravenous, oral, and topical pharmaceutical preparations (as well as in other general products including cosmetics, food, and toothpastes). The liver of an adult with normal liver and kidney functions will metabolize propylene glycol into lactate, acetate, and pyruvate within several hours [52]. Therefore, high levels of propylene glycol could be reflective of medication use, possibly in participants with liver damage or due to its simple accumulation resulting from impaired liver function. Despite the prospective nature of our study, it may be speculated that HCC cases may have encountered some symptoms, which may have prompted medical surveillance and/or alteration of dietary/lifestyle habits (e.g. reduced alcohol intake or smoking cessation). Yet, such changes would likely bias risk estimates towards the null or be unrelated to the disease outcome.

In addition to its prospective design, availability of detailed pre-diagnostic lifestyle/dietary data, and anthropometric measures, additional strengths of our study include the ability to consider liver function parameters based on a score developed from clinically relevant liver enzyme concentrations. The assumption is that decreased liver function is associated with a greater degree of liver damage. From our findings, it is apparent that the metabolic pattern associated with HCC may be reflective of liver dysfunction, as suggested by the stratified analysis on the liver function score. These results support the fact that HCC largely arises from a background of increasingly severe liver damage. Indeed, the process ending with HCC is considered to be gradual, involving infection by hepatitis viruses or the development of fatty liver diseases or cirrhosis [53]. Each part of the process may be characterized by alterations in metabolic factors, which may be detectable by metabolomic approaches [54–58]. Due to this gradual process, we note that longer follow-up time would be required in order to thoroughly assess, prior to any liver damage, the specificity of the identified HCC risk associations. Our study was composed of a large number of HCC cases that were not infected with either hepatitis B or C. Thus, we attempted to determine whether metabolomic differences could be observed in the absence of these predominant HCC risk factors. Although exclusion of hepatitis-positive cases attenuated some of our findings and resulted in loss of significance for specific metabolites, strong associations were observed for glutamate and glutamine. This is indicative of a potential defect in ammonium detoxification in non-hepatitis HCC. This observation deserves further in-depth investigation.

In our study, we were also interested in comparing metabolic changes preceding cancer diagnosis by several years. Thus, we conducted stratified analysis by lag time between blood collection and diagnosis, which showed specific metabolic changes according to follow-up time. However, a key limitation of the present study is the lack of any clinical data, assessment of any medication usage, or subgroup analyses based on pathways of HCC development. The metabolite changes associated with the later cases are more likely to be informative on the etiology and/or risk exposure (e.g. dietary components, environmental, lifestyle, and pollutants), while metabolic changes in cases diagnosed <2 years after recruitment likely reflect a direct influence of the tumor.

## Conclusion

For the first time, a metabolic pattern based on serum samples was identified to be associated with HCC risk within a large prospective study. Several metabolites associated with either an increased or decreased HCC risk have been highlighted. The majority of associations remained significant after controlling for potential confounders and consideration of correction for multiple testing. The results suggest that metabolic patterns can provide meaningful etiologic insight into HCC development and can potentially be used to detect this cancer in its early stages, even several years prior to clinical diagnosis.

## Additional file

Additional file 1:
**Supplementary methods for NMR metabolomics data acquisition, additional table (Table S1. Metabolites identified in serum samples) and additional figures (Figure S1. Validation (1000 resampling) of the O-PLS model based on 1H CPMG spectra and O-PLS metabolic signature obtained from the analysis of 1H NOESY NMR spectra.** **Figure S2.** Validation (1000 resampling) of the O-PLS models stratified by hepatitis infection status of the cases, and liver function score. **Figure S3.** Validation (1000 resampling) of the O-PLS models stratified by lag time between blood collection and diagnosis). (PDF 684 kb)

## Abbreviations

AAA: Aromatic amino acids
AFP: α-fetoprotein
ALT: Alanine aminotransferase
AP: Alkaline phosphatase
AST: Aspartate aminotransferase
AUC: Area under the curve
BCAA: Branched-chain amino-acids
CI: Confidence intervals
CLR: Conditional logistic regression
CMIAs: Chemiluminescent microparticle immunoassays
CPMG: Carr-Purcell-Meiboom-Gill
CV: Cross-validated
EPIC: European Prospective Investigation into Cancer and Nutrition
GGT: Gamma-glutamyltransferase
HBV: Chronic hepatitis B
HCC: Hepatocellular carcinoma
HCV: Chronic hepatitis C
HDL: High-density lipoproteins
LDL: Low-density lipoproteins
NMR: Nuclear magnetic resonance
NOESY: Nuclear Overhauser effect spectroscopy
O-PLS: Orthogonal partial least-square
OR: Odds ratios
ROC: Receiver operating characteristics
VLDL: Very-low-density lipoproteins
