# Supplementary material for: Metabolomic profiles of hepatocellular carcinoma in a European prospective cohort
Source: BMC Med. 2015 Sep 23;13:242. doi: 10.1186/s12916-015-0462-9 (PMC4581424; doi:10.1186/s12916-015-0462-9)
Supplement: Additional file 1: — Supplementary methods for NMR metabolomics data acquisition, additional table (Table S1. Metabolites identified in serum samples) and additional figures (Figure S1. Validation (1000 resampling) of the O-PLS model based on 1H CPMG spectra and O-PLS metabolic signature obtained from the analysis of 1H NOESY NMR spectra. Figure S2. Validation (1000 resampling) of the O-PLS models stratified by hepatitis infection status of the cases, and liver function score. Figure S3. Validation (1000 resampling) of the O-PLS models stratified by lag time between blood collection and diagnosis). (PDF 684 kb) [file 12916_2015_462_MOESM1_ESM.pdf]

Metabolomic Profiles of Hepatocellular Carcinoma in a European Prospective Cohort

This document contains :

1. Supplementary methods for the NMR data acquisition.
2. Supplementary table 1.
3. Supplementary figures 1, 2 & 3.

Supplementary methods for NMR metabolomic data acquisition

Serum sample preparation. Serum samples originally stored at IARC in cryostraws were delivered to the CRMN and were aliquoted into 200 µl tubes and stored at -80 °C until NMR profiling. Serum samples from Denmark and Sweden were directly stored at -80 °C until NMR analysis. All samples underwent the same number of freeze/thaw cycles between collection and NMR analysis. The analysis order of samples was randomized by country and matched cases and controls were analyzed consecutively in the same batch. Additionally, quality control (QC) serum samples were included to monitor the stability and reproducibility of the NMR data acquisition over the entire NMR experimental campaign as described in a previous paper (1). Before NMR data acquisition, all EPIC serum samples and QC serum samples were thawed at room temperature and processed according to standard protocols (2). In brief, 200 µl of serum was mixed with 400 µl of saline solution (0.9% NaCl in 10% D2O) and centrifuged at 4 °C, 12 000 g for 5 min. For each sample, 550 µl of supernatant was then transferred into a 5 mm NMR tube.

Spectral acquisition. NMR spectra of serum samples were acquired on a Bruker Avance III spectrometer operating at 800.15 MHz ^{1}H NMR frequency equipped with a cooled SampleJet for automatic delivery of the samples into the magnet. Samples were stored at 277 K, no more than 24 h in the SampleJet before NMR data acquisition. NMR experiments were carried out using a standard 5 mm TXI probe with the temperature regulated at 300 K. Automatic tuning and matching, frequency-locking on D_2O and 1D gradient shimming were carried out on each sample prior to data acquisition. In addition, automatic 3D gradient shimming was performed every day on a fresh QC serum sample. For proton one-dimensional NMR acquisition, the 1D Carr-Purcell-Meiboom-Gill (CPMG) pulse sequence (RD-90°-[τ-180°-τ]$_n$-FID) with water presaturation during the recycle delay was used with τ = 300 μs and n = 128 (76.8ms total echo time). For NOESY experiments, the mixing time was set to 100ms. For each experiment, 128 free induction decays (FID) were collected with 43 k data points and a spectral width of 20 ppm. The total acquisition time and the recycle delay were respectively set to 1.36 s and to 2 s. The 90° pulse length was automatically calibrated for each sample and corresponded to ~ 12 μs for 26 W. FIDs were multiplied by an exponential function, equivalent to a 0.3 Hz line-broadening factor before Fourier transformation. In addition, two-dimensional NMR experiments were recorded for a set of representative samples included ^{1}H J-resolved, ^{1}H-^{1}H TOCSY (total correlation spectroscopy) and ^{1}H-^{13}C HSQC (heteronuclear single quantum coherence) for metabolites assignment.

Processing of the spectra. ^{1}H chemical shifts of the serum spectra were automatically referenced to the anomeric proton doublet signal of α-glucose (δ 5.23 ppm). Spectra were manually phased, corrected for baseline using Topspin 3.1 (Bruker GmbH,

Rheinstetten, Germany), residual water signal (δ 4.35-5.15 ppm) and polyethylene glycol signal (δ 3.69-3.71ppm) were removed (corresponding variables set to zero) and spectra were normalized to the sum of intensities. NMR variables were Pareto scaled, i.e. each bucket was mean-centered and divided by the square root of its standard deviation, and spectra were further normalized for systematic variation introduced between the different NMR sessions (1).

1. Fages A, Pontoizeau C, Jobard E, Levy P, Bartosch B, Elena-Herrmann B. Batch profiling calibration for robust NMR metabonomic data analysis. *Analytical and Bioanalytical Chemistry.* **2013**, *405*, 8819-8827.
2. Beckonert O, Keun HC, Ebbels TMD, Bundy JG, Holmes E, Lindon JC, et al. Metabolic profiling, metabolomic and metabonomic procedures for NMR spectroscopy of urine, plasma, serum and tissue extracts. *Nature Protocols.* **2007**, *2*, 2692-2703.

Supplementary Table 1. Metabolites identified in serum samples.

Metabolite	^{1}H ppm	^{13}C ppm	Signal multiplicity	Observed by NMR
Cholesterol	0.7	14.3	m	CPMG, J-Res, TOCSY, HSQC
Lipids C**H**$_3$, HDL	0.82		m	CPMG, J-Res, TOCSY, HSQC
Lipids C**H**$_3$, LDL	0.84	16.5	m	CPMG, J-Res, TOCSY, HSQC
Lipids C**H**$_3$, VLDL	0.86	25.2	m	CPMG, J-Res, TOCSY, HSQC
Leucine	0.94	23.6	d	CPMG, J-Res, TOCSY, HSQC
Isoleucine	0.93	13.7	t	CPMG, J-Res, TOCSY, HSQC
	0.99	17.4	d	
Valine	0.98	19.9	d	CPMG, J-Res, TOCSY, HSQC
	1.02	20.7	d	
Propylene glycol	1.13		d	CPMG, J-Res
Ethanol	1.16		t	CPMG, J-Res, TOCSY, HSQC
	3.64		q	
3-hydroxybutyrate	1.20	24.5	d	CPMG, J-Res, TOCSY, HSQC
Lipids C**H**$_{2n}$, LDL	1.25	34.5	m	CPMG, J-Res, TOCSY, HSQC
Lipids C**H**$_{2n}$, VLDL	1.27	32.3	m	CPMG, J-Res, TOCSY, HSQC
Lactate	1.33	22.9	d	CPMG, J-Res, TOCSY, HSQC
	4.11	71.3	q	
Lysine	1.46		m	CPMG, J-Res, TOCSY
Alanine	1.48	19.0	d	CPMG, J-Res, TOCSY, HSQC
Lipids C**H**2-CH2-COOC	1.57	27.5	m	CPMG, J-Res, TOCSY, HSQC
Acetate	1.91	25.9	s	CPMG, J-Res, TOCSY, HSQC
Lipids C**H**2-CH=	2.01	29.8	m	CPMG, J-Res, TOCSY, HSQC
N-acetyl glycoproteins	2.02	24.8	m	CPMG, J-Res, TOCSY, HSQC
methionine	2.14	16.6	m	CPMG, J-Res
CH2-CH2-COOC	2.22	36.3	m	CPMG, J-Res, TOCSY, HSQC
Acetone	2.23	32.9	s	CPMG, J-Res, TOCSY, HSQC
acetoacetate	2.27		s	CPMG, J-Res
Glutamate	2.34		m	CPMG, J-Res, TOCSY
Pyruvate	2.37		s	CPMG, J-Res
Glutamine	2.45	33.7	m	CPMG, J-Res, TOCSY, HSQC
Citrate	2.42		d	CPMG, J-Res, TOCSY
	2.50		d	
Lipids =CH-C**H2**-CH=	2.72	28.2	m	CPMG, J-Res, TOCSY, HSQC
Albumine	2.97	42.1	m	CPMG, J-Res, TOCSY, HSQC
Creatine	3.02		s	CPMG, J-Res, TOCSY
	3.91		s	
Creatinine	3.03		s	CPMG, J-Res, TOCSY
	4.05		s	
Choline	3.19	56.6	s	CPMG, J-Res, TOCSY, HSQC
	4.07	58.4	m	
Glycerophosphocholine	3.23	56.6	s	CPMG, J-Res, TOCSY, HSQC
Phosphocholine	3.24		s	CPMG, J-Res, TOCSY
Betaine	3.27		s	CPMG, J-Res
Glycine	3.55		s	CPMG, J-Res
Glycerol	3.57	65.4	m	CPMG, J-Res, TOCSY, HSQC
Glucose	3.39	72.5	m	CPMG, J-Res, TOCSY, HSQC
	3.44	78.7	m	
	3.71	75.6	m	

	3.82	74.2	m	
	3.88	65.5	dd	
	5.22	94.9	d	
Lipids O-C**H**2	4.26	64.4	m	CPMG, J-Res, TOCSY, HSQC
Mannose	5.18		d	CPMG, J-Res
Glycerid of lipids	5.19	71.6	m	CPMG, J-Res, TOCSY, HSQC
Lipids C**H**=C**H**	5.28	130.5	m	CPMG, J-Res, TOCSY, HSQC
Tyrosine	6.87	118.6	d	CPMG, J-Res, TOCSY, HSQC
	7.17	133.4	d	
Histidine	7.75	139.1	m	CPMG, J-Res, TOCSY, HSQC
Phenylalanine	7.32	131.9	d	CPMG, J-Res, TOCSY, HSQC
	7.40		d	
Formate	8.44		s	CPMG, J-Res

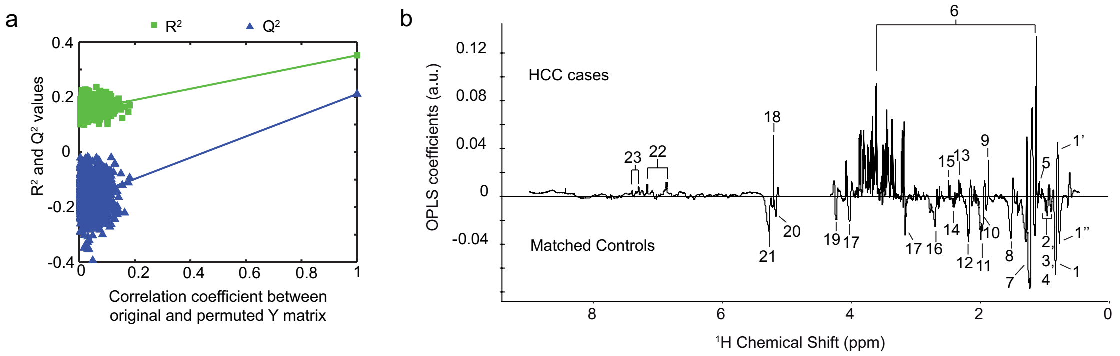

Supplementary Figure 1. (a) Validation (1000 resampling) of the O-PLS model based on ^{1}H CPMG spectra including all HCC cases (n = 114) and the matched controls (n = 222). (b) O-PLS metabolic signature obtained from the analysis of ^{1}H NOESY NMR spectra. 1, **CH**$_3$ bond of lipids mainly VLDL; 1', **CH**$_3$ bond of lipids mainly LDL; 1'', **CH**$_3$ bond of lipids mainly HDL; 2, Leucine; 3, Isoleucine; 4, Valine; 5, Propylene glycol; 6, Ethanol; 7, **CH**$_2$ bond of lipids; 8, **CH**$_2$-CH$_2$-COOC bond of lipids; 9, Acetate; 10, **CH**$_2$-CH= bond of lipids; 11, N-acetyl glycoproteins; 12, Acetone and CH$_2$-**CH**$_2$-COOC bond of lipids; 13, Glutamate; 14, Glutamine; 15, citrate; 16, =CH-**CH**$_2$-CH= bond of lipids; 17, Choline; 18, Glucose; 19, Lipid O-**CH**$_2$; 20, mannose and lipids; 21, **CH**=**CH** bond of lipids; 22, Tyrosine; 23 Phenylalanine.

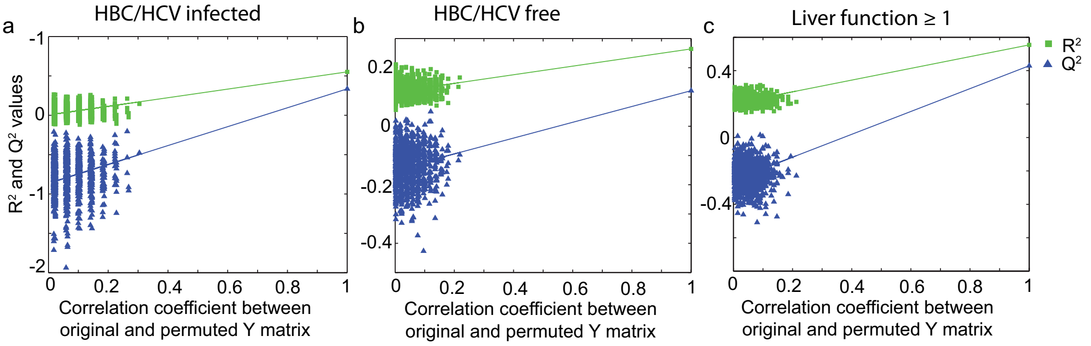

Supplementary Figure 2. Validation (1000 resampling) of the O-PLS models stratified by (a) HCC cases infected by HBV/HCV, (b) HCC cases free of hepatitis infection and (c) HCC cases with liver function score ≥ 1.

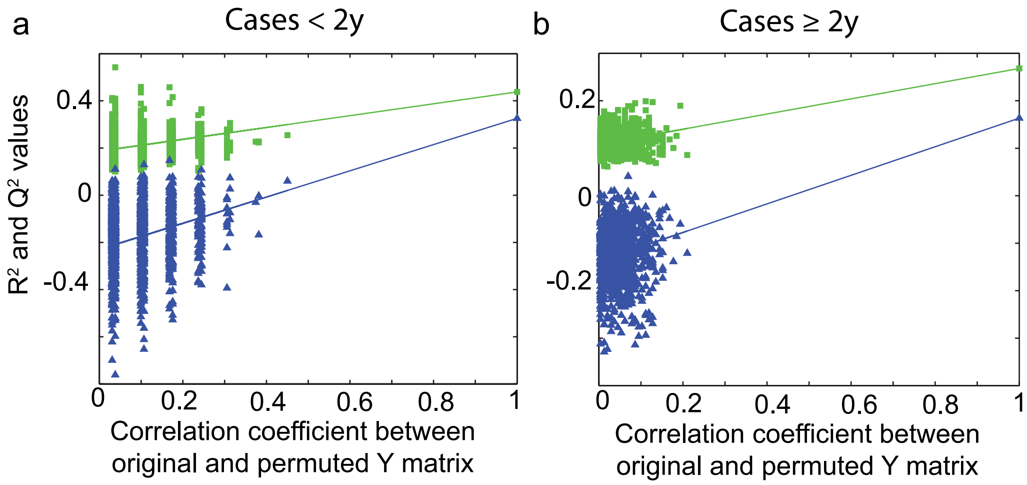

Supplementary Figure 3. Validation (1000 resampling) of the O-PLS models based on HCC cases diagnosed (a) <2 years and (b) ≥2 years after blood collection.
